# Practical Departments

**Published:** 1899-04-01

**Authors:** 


### PRACTICAL DEPARTMENTS.

### THE MATCHLESS GAS LIGHTER.

The matchless gas lighter is the name of an ingenious contrivance for lighting gas without matches. Like a great many inventions, it is the work of an Englishman, who has unfortunately been obliged to go to Germany to have it made. The agent by which the gas is ignited is named the "Lighting Pill." It is composed of a chemically treated porous block, bound round with platinum wires. This is placed close to the gas burner. When the gas is turned on, either at the meter or tap, it passes first through a "pilot igniter," a small thread-like pipe, causing the platinum wires to glow, which finally ignite the gas. The heat then generated expands a metal rod, which opens the valve of and admits the gas to the large burner. The further expansion of the rod caused by the greater heat closes the pilot valve and thus extinguishes the igniter. This arrangement may be fixed to act automatically or by pressing a button. The advantages promised by it are the prevention of all escape of gas, the shutting off all gas in the case of incandescent burners, and the preservation of mantles in incandescent burners by their gradual instead of their sudden heating. It is said to be a safer method of lighting than matches, as there is no fear of fire through carelessness.

